# The balance between intrahepatic IL-17^+ ^T cells and Foxp3^+ ^regulatory T cells plays an important role in HBV-related end-stage liver disease

**DOI:** 10.1186/1471-2172-12-47

**Published:** 2011-08-19

**Authors:** Yinghua Niu, Hongli Liu, Donglin Yin, Ruitian Yi, Tianyan Chen, Hong'an Xue, Shulin Zhang, Shumei Lin, Yingren Zhao

**Affiliations:** 1 Department of Infectious Diseases, First Affiliated Hospital of Medical College, Xi'an Jiaotong University, Shaanxi Province, China; 2 Medical College, Xi'an Jiaotong University, Shaanxi Province, China; 3 Department of Infectious Diseases, Second Affiliated Hospital of Medical College, Xi'an Jiaotong University, Shaanxi Province, China

## Abstract

**Backgroud:**

IL-17^+ ^T helper cells and Foxp3^+ ^regulatory T cells are CD4^+ ^T helper cells with reciprocally regulated differentiation and function. Their frequency and function vary in patients with chronic hepatitis B. In this study, we investigated the balance between IL-17^+ ^T cells and Foxp3^+ ^regulatory T cells and illustrated their function in the aggravation of chronic hepatitis B (CHB).

**Results:**

Twenty-six patients with chronic HBV -related liver failure (CLF), thirty-one patients with acute on chronic HBV-related liver failure (ACLF) and twelve normal controls were enrolled in our study. The expressions of IL-17, Foxp3, CD4, CD8 and perforin in liver tissue were measured by immunochemistry for the evaluation of liver-infiltrating lymphocytes. The frequency of liver IL-17^+ ^T cells on liver inflammatory cells and their proportion in the total CD4^+ ^T cell population increased markedly in the ACLF group, while the frquency of Foxp3^+ ^T cells and their proportion in the total CD4^+ ^T cell population did not show a significant difference in the two HBV infection groups. In addition, the ACLF group showed a dramatically higher IL-17^+ ^/Foxp3^+ ^ratio than the CLF group. CD4^+ ^T cells increased significantly in the liver of patients with ACLF, compared with those in the liver of patients with CLF.

**Conclusions:**

Our findings suggest that intrahepatic IL-17^+ ^T cells play an important role in the development of chronic HBV and that the imbalance between IL-17^+ ^and Foxp3^+ ^T cells in the liver may lead to progression of the disease but the mechanism should be further explored.

## Background

Chronic hepatitis B (CHB) infection affects over 350 million individuals worldwide. It has become a global health problem due to its manifestation as chronic liver failure (CLF), acute on chronic liver failure (ACLF), liver cirrhosis (LC), and primary hepatocellular carcinoma (HCC)[1,2]. Hepatitis B virus (HBV) is preferentially hepatotropic, not directly cytopathic, and elicits liver diseases of different severity [3]. HBV may also cause sustained liver tissue damage through different pathways, including perforin-mediated cytotoxicity and Fas ligand/Fas-mediated apoptosis, when antiviral immunity is not vigorous enough to clear the virus [4]. CD8 ^+ ^T cells are the main effector cells for the elimination of HBV [5]. Thus, the hepatocellular injuries caused by HBV infection are predominantly immune-mediated [1,2]. Studies investigating the frequency of intrahepatic virus-specific CD8^+ ^T cells have shown that the non-virus-specific infiltrating CD8^+^T lymphocytes participate in liver damage in HBV infection [6]. The CD4/CD8 T cell ratio is 1:3.5 for the liver versus 2:1 for blood lymphocytes [7], suggesting that the intrahepatic CD4 ^+ ^and CD8 ^+ ^T cells may have a specialized role in the pathogenesis of liver disease through an immune response.

CD4 ^+ ^T helper cells perform critical immune functions via the production of distinct cytokine profiles. Two new subsets of CD4 ^+ ^T cells, IL-17^+ ^T helper cells (Th17) and Foxp3^+ ^regulatory T cells (Treg), have been described [8,9]. Evidence has shown that circulating IL-17^+ ^cells are largely accumulated in the livers of CHB patients and that their frequency increases with progression from CHB to ACLF [10,11]. It has been reported that the increased levels of Treg cells in the blood and liver of CHB patients correlated with suppression of HBV antigen-specific T cell effects or responses *in vitro *[12]. Treg cells play a part in the control of chronic inflammatory responses and contribute to pathologic events in the liver during HBV infection. Th17 cells are implicated in host defense against a number of microorganisms [13,14], while Treg cells display a suppressive function in immune responses and inflammatory diseases [15,16]. A balance between Th17 and Treg cells is crucial for immune homeostasis. However, the variations in Th17 and Treg cells in the progression of HBV- related liver failure is still not clear.

Here we considered whether the frequency and ratio of intrahepatic Th17 and Treg cells were changed or disordered during the development of HBV-related ACLF. In this work, we investigated the frequency of IL-17^+ ^and Foxp3^+ ^T cells in the liver tissue of patients with HBV-related ACLF and CLF by immunochemistry and analyzed the possible association between the frequencies of IL-17 and Foxp3 positive cells with various clinical parameters, aiming to understand the mechanism underlying the effects of Th17/Treg cells on the development of ACLF.

## Results

### Clinical data of the three groups

Table 1 shows the clinical data of the CLF, ACLF and NC groups. There were more male than female patients in the three groups. Patients in the HBV-related CLF and ACLF groups were younger than those in the NC group. There was a clear increase in the alanine aminotransferase (ALT), total bilirubin (Tbil), international normalized ratio(INR) and MELD score in the CLF and ACLF groups, compared with those in the NC group. The levels of Tbil, INR and MELD score were even higher in the ACLF group than those in the CLF group (*p *= 0.0001, all). The cholesterol (CHOL) level in the ACLF group was lower than that in the CLF group (p = 0.0001). The hepatitis B e antigen (HBeAg) positive rate and hepatitis B deoxyribonucleic acid load (HBV DNA load) were almost the same in the CLF and ACLF groups (*p *= 0.225 and 0.678, respectively).

**Table 1:** Clinical characteristics of the subjects

				*P value*		
Group(N)	ACLF(31)	CLF(26)	NC(12)	ACLF vs CLF	ACLF vs NC	CLF vs NC
Gender(m/f)	28/3	22/4	7/5			
Agea	40.0(5.5)	47.5(7.5)	48(4.75)	.039	.013	.694
ALT(IU/L) ^a^	93(70.5)	45.5(26.8)	19.0(4.87)	.061	.00	.000
Tbil(μmol/L) ^a^	303.4(201.8)	37.15(21.44)	16.4(1.91)	.000	.000	.001
CHOL (mmol/L) ^a^	1.01(0.63)	2.2(0.3)	3.8(0.76)	.000	.000	.000
INR^a^	2.50(0.48)	1.63(0.34)	0.96(0.06)	.000	.000	.000
HBeAg (+/-)	9/22	4/22	NT	.225		
HBVDNA(+/-)	23/8	18/8	NT	.679		
MELD score^a^	26.49(3.76)	11.39(3.84)	0(0)	.000	.000	.000

NT, not detected; ALT, alanine aminotransferase; CHOL, cholesterol; Tbil, total bilirubin; INR, international normalized ratio; HBeAg, hepatitis B e antigen; anti-HBe, hepatitis B e antibody; HBVDNA, hepatitis B deoxyribonucleic acid load; MELD, Model for End-stage Liver Disease.

^a^The medians (interquartile range) of age, ALT, Tbil, CHOL, INR and MELD for each group are shown

### Distribution of IL-17^+ ^and Foxp3^+ ^T cells in liver tissues

**Figure 1:**
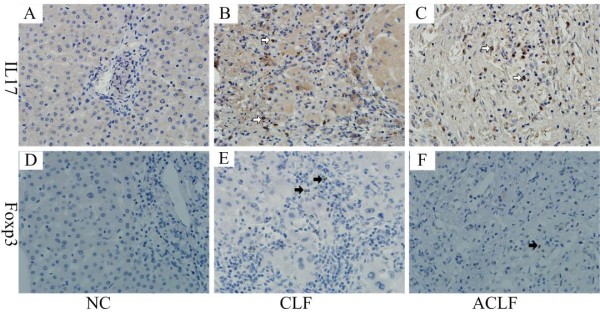
**Liver infiltration of IL17^+ ^and Foxp3^+ ^T cells**. Foxp3 (→) was distinctly expressed in the nuclei of lymphocytes (D: NC, E: CLF, F: ACLF) (×400) while IL-17 (→) showed granular cytoplastic staining (A: NC, B: CLF, C: ACLF) (×400). A large number of lymphocytes infiltrated into the livers of the CLF and ACLF patients. IL17**^+ ^**and Foxp3**^+ ^**T cells were mainly located in the portal area of the liver and could still be found in the lobe, especially in the necrotic area (C). NC, normal control; CLF, HBV-related chronic liver failure; ACLF, HBV-related acute on chronic liver failure.

Immunochemistry staining of IL-17 and Foxp3 in the liver tissue of the three groups was performed to study whether the frequencies of IL-17^+ ^and Foxp3^+ ^T cells and the IL-17^+ ^/Foxp3^+ ^ratio varied during the course of HBV-related ACLF. Foxp3 was distinctly expressed in the nuclei of the T lymphocytes while IL-17 showed granular cytoplasmic staining (Figure 1). As shown in Table 2, a large number of lymphocytes infiltrated the liver of patients with HBV-related CLF and ACLF. IL-17^+ ^T cells were mainly located in the portal area of the liver and could still be found in the lobes, especially in the necrotic area (Figure 1C). A significantly strong expression of IL-17^+ ^T cells was detected in the ACLF group (median = 3.18%, interquartile range = 2.31%), compared with that in the CLF group (median = 1.90%, interquartile range = 0.84%; *p *= 0.0001). The Foxp3^+ ^T cells mainly infiltrated the portal area of the liver (Figure 1E and 1F) in the CLF and ACLF groups and were evidently increased compared to the NC group, but there were no significant differences between ACLF and CLF groups. The IL-17^+ ^/Foxp3^+ ^ratio was higher in the ACLF group than in the CLF group (*p *= 0.004).

**Table 2:** The medians of parameters and statistic data in the three groups

				*P value*		
Group(N)	ACLF(31)^a^	CLF(26)^a^	NC(12)^a^	ACLF vs CLF	ACLF vs NC	CLF vs NC
IL-17(%)	3.18(2.31)	1.90(0.84)	0(1.41)	.000	.000	.000
FoxP3 (%)	0.36(0.55)	0.23(0.44)	0.00(1.42)	.621	.000	.000
IL17/Foxp3	7.00(10.30)	4.33(4.66)	0.00(1.5)	.004	.000	.002
CD4 (%)	60.94(8.77)	57.24(15.06)	45.84(16.22)	.060	.000	.000
IL-17/CD4(%)	5.32(4.51)	3.49(2.11)	0(4.92)	.008	.001	.019
FoxP3/CD4(%)	0.62(0.93)	0.69(1.06)	1.35(5.11)	.215	.937	.577
CD8 (%)	35.90(8.82)	38.71(14.91)	46.38(15.47)	.054	.000	.000
Perforin (%)	7.50(4.35)	5.16(3.32)	2.03(4.17)	.086	.002	.003

The proportion of positively stained cells on the liver inflammatory cells in five randomly-selected high power fields (hpf, ×400) were analyzed by two independent observers who were blinded to the clinical data of the liver tissues.

^a ^The frequency and ratio of positive cells in the three groups are expressed as medians (interquartile range).

NC, normal control; CLF, HBV-related chronic liver failure; ACLF, HBV-related acute on chronic liver failure.

The CD4^+ ^T lymphocytes in the liver tissue of the three groups were stained to determination of the percentages of IL-17^+ ^and Foxp3^+ ^T cells in CD4^+ ^T cells. The ACLF and CLF patients had a higher IL-17^+^/CD4^+ ^ratio than the NC group, and the IL-17^+^/CD4^+ ^ratio was remarkably higher in the ACLF group than in the CLF group and NC groups (*p *= 0.008 and 0.001, respectively). The Foxp3^+^/CD4^+ ^ratio increased slightly in the CLF and ACLF groups but the differences between the three groups were not statistically significant.

### Distribution of CD4^+ ^T cells, CD8^+ ^T cells and perforin^+ ^cells in liver tissues

**Figure 2:**
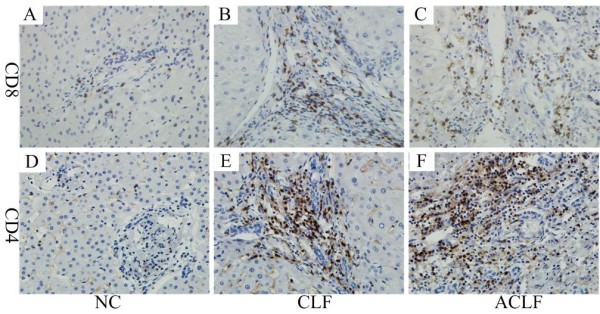
**Liver infiltration of CD4^+ ^T cells and CD8^+ ^T cells**. CD8 (A: NC, B: CLF, C: ACLF) and CD4 (D: NC, E: CLF, F: ACLF) (×400) showed diffuse non-granular cytoplasmic staining while perforin showed granular cytoplasmic staining. The positive cells mainly infiltrated into the portal areas of the liver. NC, normal control; CLF, HBV-related chronic liver failure; ACLF, HBV-related acute on chronic liver failure.

**Figure 3:**
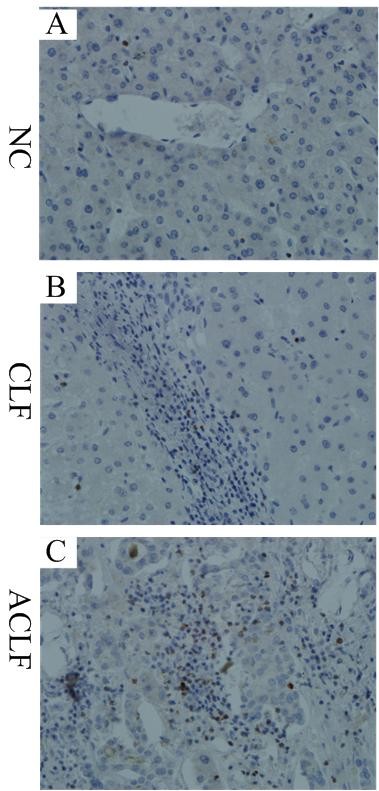
**Liver infiltration of perforin^+ ^cells**. Perforin showed granular cytoplastic staining (A: NC, B: CLF, C: ACLF) (×400). NC, normal control; CLF, HBV-related chronic liver failure; ACLF, HBV-related acute on chronic liver failure.

The staining of CD4, CD8 and perforin was performed to determine the levels of CD4^+ ^T cells, CD8 ^+ ^T cells and perforin^+ ^cells in the liver. CD4 and CD8 staining showed a diffuse non-granular cytoplasmic staining pattern while perforin showed a granular cytoplastic staining pattern (Figures 2, 3). The frequencies of CD4^+ ^T cells and perforin^+ ^cells were significantly increased in the liver of patients with CLF and ACLF, compared with those in the NC group. But the frequency of CD8^+ ^T cells was significantly decreased in the liver of patients with CLF and ACLF, compared with those in the NC group. The positive cells mainly infiltrated the portal areas of the liver.

The frequencies of CD4^+ ^T cells and perforin^+ ^cells were higher in the ACLF patients than those in the CLF patients but with no statistical difference (*p *= 0.060 and *p *= 0.086, respectively). On the other hand, there were more CD8^+ ^T cells in CLF than that in ACLF group but with no statistical difference (*p *= 0.054).

### Correlation between the frequencies of specific positive cells and clinical parameters

To explore the possible role of liver infiltrating IL-17^+ ^and Foxp3^+ ^T cells in the development of HBV-related liver failure, we further analyzed the correlation between the frequencies of specific positive cells and clinical laboratory test parameters of the three groups using Spearman's rank correlation analysis. Among the most commonly used clinical data for patients with HBV infection, serum HBV load usually represents virus replication, and serum ALT levels reflect hepatocellular inflammatory injury. In addition, Tbil and CHOL are important indexes for measuring the severity of hepatic injury, and the MELD score is widely used to estimate liver function in end-stage liver disease.

**Figure 4:**
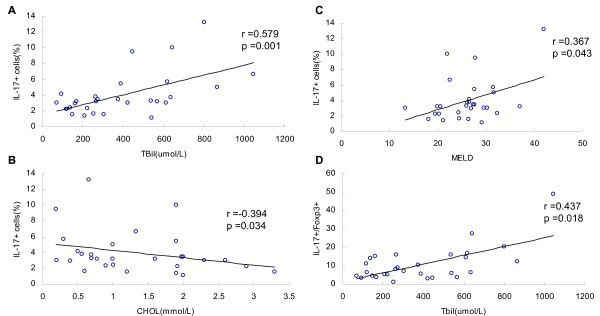
**Correlation between the frequency of IL17^+ ^cells, the ratio of IL17+/Foxp3+ and the clinical parameters**. The frequency of IL17**^+ ^**T cells correlated with Tbil (A, *r *= 0.579, *p *= 0.001), CHOL (B, *r *= -0.394,*p *= 0.034) and MELD scores (C, *r *= 0.367, *p *= 0.043), and the ratio of IL17+/Foxp3+ correlated with Tbil (D, *r *= 0.437, *p *= 0.018).

In the ACLF group, the frequency of IL17^+ ^T cells correlated with Tbil (*r *= 0.579, *p *= 0.001), CHOL (*r *= -0.394, *p *= 0.034) and MELD score (*r *= 0.367, *p *= 0.043), and the ratio of IL17^+^/Foxp3^+ ^correlated with Tbil (*r *= 0.437, *p *= 0.018) (Figure 4). The frequency of CD8^+ ^T cells showed no correlation with Tbil and MELD scores (*r *= -0.067 and -0.28, respectively). The frequency of perforin^+ ^cells correlated with Tbil (*r *= 0.443, *p *= 0.023).

## Discussion

IL-17 is mainly produced by a distinct subset of CD4^+ ^T helper cells called Th17 cells. IL-17 may be a new marker for the severity of acute hepatic injury since levels increase in patients with severe acute hepatic injury or fulminant hepatic failure [17]. Liver infiltration of IL-17^+ ^T cells has also been found to be positively associated with the grade of liver inflammation in CHB patients [11]. In fact, most experimental evidence to date suggests a role for IL-17 family members in the coordination of local tissue inflammation, mainly via the induced release of pro-inflammatory and neutrophil-mobilizing cytokines [18]. It has been found that the frequency of circulating IL-17^+ ^T cells increases with disease progression from CHB to ACLF [10].

To understand whether the frequency of intrahepatic IL-17^+ ^T cells increases during the course of HBV-related ACLF just as the frequency of circulating IL-17^+ ^T cells does, we performed immunochemistry staining of IL-17 and CD4 in liver biopsies from 57 patients with HBV related end-stage liver diseases. The frequency of intrahepatic IL-17^+ ^T cells and the IL-17^+^/CD4^+ ^ratio increased significantly in HBV-related ACLF and CLF patients, and the increase in IL-17^+ ^T cells positively correlated with Tbil, CHOL and MELD score. Therefore, the increase in IL-17^+ ^T cells can reflect the grade of hepatic injury during the progression of liver disease. Inappropriate, excessive, or non-specific IL-17^+ ^T cell effector responses may be involved in the pathogenesis of HBV-related ACLF. However, little is known about the regulatory role of IL-17^+ ^T cells in HBV infection. An *in vitro *study by Li, J et al. has demonstrated that HBcAg can stimulate the production of IL-10, which negatively regulates HBcAg-specific Th17 cell responses in CHB patients [19]. Similar findings in a study of chronic hepatitis C virus (HCV) has also revealed that HCV-specific Th1 and Th17 cells are suppressed by HCV nonstructural protein 4 (NS4) -induced production of IL-10 and transforming growth factor (TGF)-β [20].

Th17 and Treg, which are two subsets of CD4^+^T cells, share reciprocal developmental pathways in immune responses. The generation of Treg cells is dependent on a critical differentiation factor named TGF-β [21]. An experiment in mice demonstrated that the generation of Foxp3^+ ^Treg cells can be completely inhibited by IL-6 induced during inflammation, while the differentiation of pathogenic Th17 cells from naive T cells is induced by IL-6 plus TGF-β[22]. Notably, a more recent study has suggested that Th17 cells play a crucial role in the mediation of airway inflammatory responses and that antigen-specific Treg cells suppress Th17-mediated lung inflammation [23].

Immunochemistry staining of Foxp3 was used to determine the number of Treg cells infiltrating the liver tissue since Foxp3 is not only a transcription factor but also a specific marker for Treg [24]. Our result showed that the frequency of Foxp3^+ ^T cells increased significantly in HBV-related ACLF and CLF patients compared to NC, but there was no significant difference between the CLF and ACLF patients. Xu et al. have found that the frequency of Foxp3^+ ^Treg cells increased dramatically in the circulation and liver of 9 patients with chronic severe hepatitis B, and that the increase in Treg at the inflammatory site is associated with the chronicity and severity of liver inflammation [12]. Our finding that Foxp3^+ ^T cells in the ACLF and CLF patients were similar is in contrast to that study, probably because the patients enrolled in our study suffered from severe and end-stage liver diseases. As a result, there was a small increase in Foxp3^+ ^T cells but they were unable to suppress the inflammation, leading to massive hepatic necrosis and thereafter, to poor prognosis.

The IL-17^+^/Foxp3^+ ^ratio in the ACLF patients was higher than that in CLF patients, which suggests that the number of IL-17^+ ^T cells increased and caused immune hepatic injury in the progression of ACLF. However, although the numbers of suppressive Foxp3^+ ^T cells increased a little, they did not reach the same proportion as the IL17^+ ^T cells, leading to a relative shortage of suppressive factors. This suggests that the imbalance of the IL-17^+^/Foxp3^+ ^ratio in the liver infiltrating lymphocytes may be the key factor for the development of fatal ACLF.

CD8^+ ^T cells participate in the elimination of HBV and may cause sustained liver damage [5] while Treg cells have a negative regulatory function. Previous studies on chronic hepatitis C have found that human CD4^+ ^CD25^+ ^T cells can cause pronounced and sustained inhibition of CD8^+ ^T cell proliferation [25,26]. Franzese et al [27] have found that the frequencies of CD4^+^CD25^+^Treg cells showed no significant difference in patients with immunotolerant, chronic active and asymptomatic HBV infections, while the decrease in CD4^+ ^CD25^+ ^T cell frequency was found in patients with a flare-up of chronic hepatitis B. An *in vitro *study showed that depletion of the CD4^+ ^CD25^+ ^T cell population affected not only the expansion of HBV-specific CD8^+ ^T cells but also their function [27]. This confirms the ability of circulating CD4^+ ^CD25^+ ^T cells to suppress antiviral immune responses mediated by CD8^+ ^T cells [25,28]. CD4^+ ^CD25^+ ^T cells are activated to suppress the expansion of HBV-specific CD8^+ ^T cells, thus precluding HBV clearance but limiting excessive immune-mediated liver damage. This regulation is clearly non-antigen specific [27].

## Conclusions

Our study suggests that intrahepatic IL17^+ ^T cells played an important role in the development of chronic hepatitis B and that an imbalance between IL-17^+ ^and Foxp3^+ ^T cells in the liver might lead to progression of disease. Because the increase in Foxp3^+ ^T cells in the liver-infiltrating lymphocytes of ACLF and CLF patients was not proportional with the increase in IL-17^+ ^T cells, the Foxp3^+ ^T cells could not negatively regulate the immune system and ultimately, this would cause liver failure. We did not study the frequencies of T cell subsets infiltrating into the liver by using more precise methods such as flow cytometry or study the proportions of these subsets in the peripheral blood simultaneously or dynamically. Thus, we do not know the exact mechanism underlying the effects of the Treg cells on the Th17 cells and CD8^+ ^T cells. The function of IL-17^+ ^T cells and Foxp3^+ ^T cells in the progression of HBV-related ACLF should be further explored.

## Methods

### Subjects

We recruited 57 patients with end-stage liver disease following liver transplantation at the First Affiliated Hospital of Medical College, Xi'an Jiaotong University (Xi'an, China) from January 2002 to June 2009. Patients were classified into two groups. Twenty-six patients suffered from HBV-related CLF and 31 patients were diagnosed with HBV-related ACLF according to the corresponding diagnostic criteria [29]. We excluded patients with comorbidities such as hepatitis C infection, rheumatoid arthritis, autoimmune hepatitis and hepatocellular carcinoma according to their clinical information and histological data. Twelve patients who had undergone hepatic hemangioma surgery and had normal liver tissues were used as normal controls (NC). Patients with viral hepatitis, autoimmune hepatitis and alcoholic liver diseases were excluded from the normal controls. The institutional review board of the First Affiliated Hospital of Medical College, Xi'an Jiaotong University approved these protocols, and informed consent was obtained from all individuals.

Clinical data of the patients were recorded in detail and summarized in Table 1. The Model for End-stage Liver Disease (MELD) score was calculated by the formula: 3.8*log_e_(bilirubin [mg/dL])+ 11.2*log_e_(INR) + 9.6*log_e_(creatinine [mg/dL]) +6.4*(etiology: 0 if cholestatic or alcoholic, 1 otherwise) [30].

### Immunohistochemical staining

Paraffin-embedded, formalin-fixed liver tissues were cut into 5-μm sections and placed on polylysine-coated slides. Each paraffin section was deparaffinized and rehydrated through a graded series of ethanol. The antigen retrieval (Table 3) was performed by steaming the slides in appropriate buffer at different temperatures and pressures. Endogenous peroxidase was blocked using a 3%H_2_O_2 _methanol solution. 5% bovine serum albumin was applied to block nonspecific staining. Rat mAbs for anti-human Foxp3 (eBiosciences, San Diego, CA), rabbit anti-human IL-17 (Beijing Bioss Biotech, Beijing, China), rabbit anti-human CD4, CD8 and mouse anti-perforin (Zymed, San Diego, CA) were used for the immunochemistry staining of Foxp3, IL-17, CD4, CD8 and perforin, respectively. Incubation of biotin-free secondary antibody and horseradish peroxidase (Zhongshan Goldenbridge Biotech, Beijing, China) was subsequently performed, followed by development with diaminobenzidine (Sigma, St Louis, MO, USA) and counterstaining with hematoxylin. Negative control staining was performed with cold PBS, instead of the primary antibody.

**Table 3:** Primary antibodies

Antigen	dilution radio	antigen retrieval	source	Temperature of incubation of primary antibody	Time of incubation of primary antibody	Time of DAB	Time of staining with hematoxylin
CD4	1:20	citrate buffer solution pressure cooking (pH 6.0) 2 min	Rabbit	37°C	1 hr	6 min	5 min
IL-17	1:100	Tris-EDTA(pH 9.0) 15 min	Rabbit	37°C	1 hr	8 min	5 min
Foxp3	1:100	Tris-EDTA(pH9.0) 15 min	Rat	37°C	1 hr	10 min	2 min
CD8	1:50	Citrate buffer solution pressure cooking(pH 6.0) 2 min	Rabbit	4°C	Overnight	8 min	3 min
perforin	1:10	EDTA(pH 8.0) pressure cooking 2 min	Mouse	4°C	Overnight	8 min	3 min

### Data collection of Immunohistochemical staining

Two experienced pathologists separately counted the number of positive staining in 5 randomly chosen (×400) high-power fields of view. And the numbers of the total liver inflammatory cells in the same 5 high-power fields of view were also counted. Then the frequency of each positive staining on the total liver inflammatory cells was got. Each pathologist was unaware of the clinical diagnosis of the patient associated with any of the tissue sections at the time of analysis.

### Statistical analysis

Results were expressed as medians (interquartile range). Statistical comparisons between two groups were made by a Mann-Whitney non-parametric U test. Spearman's correlation analysis was performed to evaluate the relationship between two variables. Data were analyzed using SPSS version 16.0 for Windows (SPSS Inc., Chicago, IL). P values of < 0.05 were considered significant for all analyses.

## Competing interests

The authors declare that they have no competing interests.

## Authors' contributions

YHN carried out all the experiments, analyzed results and drafted the manuscript. HLL helped to edit the manuscript. Some help was given by DLY, RTY and YRZ in analysis of data and preparation of the manuscript. TYC, SLZ, HAX and SML participated in the design of the study and critically reviewed the manuscript. All authors read and approved the final manuscript.
